# Author Correction: Identification of Novel Clinically Relevant Variants in 70 Southern Chinese patients with Thoracic Aortic Aneurysm and Dissection by Next-generation Sequencing

**DOI:** 10.1038/s41598-020-66839-4

**Published:** 2020-06-12

**Authors:** Miaoxian Fang, Changjiang Yu, Siyao Chen, Weiping Xiong, Xin Li, Rong Zeng, Jian Zhuang, Ruixin Fan

**Affiliations:** 1 Department of Intensive Care Unit of Cardiac Surgery, Guangdong Cardiovascular Institute, Guangdong General Hospital, Guangdong Academy of Medical Sciences, Guangdong, China; 2 Department of Cardiac Surgery, Guangdong Cardiovascular Institute, Guangdong General Hospital, Guangdong Academy of Medical Sciences, Guangdong, China

Correction to: *Scientific Reports* 10.1038/s41598-017-09785-y, published online 30 August 2017

This Article contains typographical errors in Table 6.

The reference sequences of ACTA2 and MYH11, ‘NM_001141945’ and ‘NM_001040114’ were incorrectly given as ‘NM_001613’ and ‘NM_002474’, respectively.

